# Neural mechanisms of acupuncture in amnestic mild cognitive impairment: a protocol for an fMRI-based systematic review and meta-analysis

**DOI:** 10.3389/fneur.2025.1641297

**Published:** 2026-01-02

**Authors:** Tao Zhu, Dan Yang, Fei Quan, Yizhou Chen, Jin Cui

**Affiliations:** 1 School of Acupuncture and Tuina, Guizhou University of Traditional Chinese Medicine, Guiyang, China; 2 Department of Rehabilitation Medicine, The First Affiliated Hospital of Guizhou University of Traditional Chinese Medicine, Guiyang, China; 3 Department of Acupuncture, The First Affiliated Hospital of Guizhou University of Traditional Chinese Medicine, Guiyang, China

**Keywords:** acupuncture, meta-analysis, mild cognitive impairment, neural effects, protocol, systematic review

## Abstract

**Background:**

Amnestic mild cognitive impairment (aMCI), characterized by progressive memory decline, represents a prevalent transitional state in global aging populations and exhibits high conversion rates to Alzheimer’s disease (AD), constituting a critical window for preventive interventions. While accumulating evidence supports acupuncture’s efficacy in enhancing cognitive performance, the precise neural mechanisms underlying its therapeutic effects remain poorly characterized. This neuroimaging investigation aims to elucidate the cerebral reorganization patterns mediating acupuncture-induced cognitive improvement in aMCI pathophysiology.

**Methods and analysis:**

A systematic search strategy was implemented across eight electronic databases supplemented by manual searches, covering publications from each database’s inception to May 1, 2025. Eligible study designs included randomized controlled trials (RCTs), prospective case–control studies, and observational investigations. Two independent investigators performed literature screening and data extraction, with discrepancies resolved through consensus or third-party adjudication. Methodological quality appraisal was conducted using the validated Agency for Healthcare Research and Quality (AHRQ) checklist. Primary outcomes focused on resting-state functional MRI (rs-fMRI) whole-brain functional imaging parameters. Meta-analytic synthesis of neuroimaging data will utilize seed-based d mapping with permutation of subject images (SDM-PSI, version 6.21), while clinical outcome analyses will be performed using RevMan 5.3 software (Cochrane Collaboration). Reporting will strictly adhere to PRISMA (Preferred Reporting Items for Systematic Reviews and Meta-Analyses) guidelines.

**Conclusion:**

This study synthesizes findings from independent neuroimaging investigations to establish comprehensive evidence supporting the neurotherapeutic effects of acupuncture in aMCI.

**Systematic review registration:**

https://www.crd.york.ac.uk/prospero/, Identifier CRD420251033511.

## 1 Introduction

Mild cognitive impairment (MCI) represents a prevalent neurological disorder characterized by cognitive deficits that exceed the expected decline associated with normal aging, yet do not meet the criteria for dementia due to the absence of significant impairment in daily functioning (1). Amnestic mild cognitive impairment (aMCI), a predominant subtype of MCI, is clinically characterized by measurable impairments in episodic memory and progressive cognitive decline (1). The aMCI is primarily characterized by memory decline and increased frequency of forgetting events, whose associated risks are often overlooked. A 2022 meta-analysis synthesizing global research data revealed a MCI prevalence of 15.56% in individuals aged 50 and older, with the prevalence demonstrating a positive correlation with advancing age, consistent with these findings (2). Two studies focusing on the Chinese population have reported consistent findings: middle-aged and older adults exhibit a high prevalence of cognitive impairment. Furthermore, these studies observed elevated prevalence rates among women, rural residents, individuals living alone, and those with lower educational attainment compared to other demographic groups (3, 4). The clinical progression trajectory of Alzheimer’s disease (AD) comprises three consecutive stages: the preclinical stage, MCI stage, and dementia stage. Among these continuum, MCI demonstrates the highest prevalence rates and predominantly manifests as amnestic memory impairment. As such, the aMCI phase is widely regarded as the prodromal trajectory of Alzheimer’s disease progression, while simultaneously representing a critical yet frequently overlooked window for preventive intervention in affected individuals (5). The scientific community recognizes the aMCI phase as the critical window for AD prevention, where early diagnosis and therapeutic intervention have been positioned as a clinical priority to delay dementia progression, with potential to mitigate AD pathogenesis. To date, no systematic evidence has confirmed the efficacy of any intervention for memory impairment and neuropsychological symptoms in aMCI (6). Although first-line pharmacological treatments can provide transient cognitive improvement, the early nature of memory impairment is frequently overlooked in community populations, and their clinical utility remains debated when considering the risk–benefit profile of their adverse effects (7). Consequently, exploring effective treatment strategies to address memory-predominant cognitive deficits and reduce disease burden has become a critical and urgent priority in clinical research.

Acupuncture, a traditional Chinese medical technique characterized by the insertion of fine needles into cutaneous and deeper anatomical structures, has demonstrated significant therapeutic efficacy in enhancing cognitive performance (8–10). A meta-analysis has demonstrated that electroacupuncture improves cognitive function and represents an effective therapeutic approach for MCI (8). Another acupuncture study has demonstrated that auricular vagus nerve stimulation effectively improves cognitive function in patients with MCI, with favorable clinical efficacy and safety profiles (11). Acupuncture research on cognitive enhancement has accumulated substantial evidence in recent years, with established efficacy in ameliorating cognitive deficits among middle-aged and elderly populations and delaying progression from aMCI to dementia (12–15).

Resting-state functional magnetic resonance imaging (rs-fMRI) is a neuroimaging modality characterized by non-invasive operation and absence of ionizing radiation, which evaluates regional cerebral function through detecting spontaneous fluctuations in blood oxygen level-dependent (BOLD) signals (16). With advancements in fMRI technology, this neuroimaging modality has been increasingly implemented in cognitive studies involving acupuncture to elucidate the associated neurophysiological mechanisms (17). Investigators have conducted comparative analyses of Taixi (KI3) acupoint and non-meridian, non-acupoint interventions, revealing that Taixi acupuncture evokes differential amplitude of low-frequency fluctuation (ALFF) alterations across cerebral regions, suggesting distinct regulatory effects of specific acupoints on neural network modulation (18). Emerging evidence has indicated that acupuncture enhances functional activity in specific cerebral regions and improves cognitive performance, with key regions implicated including the parahippocampal gyrus, precuneus, and cingulate cortex (19). Current studies have yielded inconsistent findings, highlighting that the neurofunctional mechanisms underlying acupuncture intervention for aMCI remain incompletely elucidated, thereby warranting rigorous investigation into its cerebral neuromodulatory effects on cognitive networks.

To date, systematic reviews or meta-analyses have been conducted to comprehensively analyze fMRI data in acupuncture treatment for MCI (20). Unfortunately, although this critical phase represents a golden window for dementia prevention, neuroimaging analyses specifically focusing on memory impairment and elucidating the neural mechanisms underlying memory alterations remain absent. This gap exists because progressive memory impairment is the symptom most frequently overlooked by MCI patients in daily life, as memory decline is often misattributed to normal aging—which is generally not the case. Conducting dedicated neuroimaging analyses targeting the neural mechanisms of aMCI, aligned with this clinical characteristic, would advance understanding of the memory domain in early-stage cognitive impairment. Therefore, to clarify the role of specific brain regions in acupuncture-mediated improvement of cognitive-memory functions, we integrated published neuroimaging studies on acupuncture for aMCI, analyzed its efficacy and safety in treating memory-predominant cognitive impairment, and performed a quantitative meta-analysis of its clinical advantages and effects on memory-related brain regions. This work establishes a solid foundation for developing clinical efficacy prediction models that select and activate dominant brain regions to improve memory impairment, thereby supporting clinicians in making better-informed decisions for their patients.

## 2 Method

### 2.1 Research registration

This protocol has been registered in the PROSPERO International Register of Prospective Systematic Reviews (Registration Number: CRD420251033511). All reporting procedures have been strictly implemented adhere to the Preferred Reporting Items for Systematic Reviews and Meta-Analyses (PRISMA) reporting guidelines (21).

### 2.2 Patient and public involvement

No patients or public are involved in the design, conduct, reporting or dissemination of this research.

### 2.3 Literature search

A comprehensive search of the following eight databases will be conducted in August 2025: PubMed, Embase, Web of Science, the Cochrane Library, China National Knowledge Infrastructure (CNKI), VIP Information Database, Wanfang Data, and China Biomedical Literature Database (CBM). The literature search spanned from the inception of each database to May 1, 2025. Publications were restricted to English and Chinese languages. The search strategy incorporated Medical Subject Headings (MeSH) combined with free-text terms, specifically targeting aMCI, acupuncture, and fMRI, with Boolean logic applied using “OR” and “AND” operators. Table 1 details the PubMed search strategy. Additionally, reference lists of all included studies, relevant clinical trial reports, and review articles will be manually screened. Manual searches will be performed in specialized journals, bibliographies, and conference proceedings related to aMCI and acupuncture available in library collections. Furthermore, comprehensive searches will be conducted in the WHO International Clinical Trials Registry Platform (ICTRP) and the Chinese Clinical Trial Registry (ChiCTR) to identify ongoing or unpublished trials, thereby ensuring the methodological comprehensiveness of the literature search.

**Table 1:** The search strategy for PubMed.

Order	Strategy
#1	“Mild cognitive impairment”[Mesh]
#2	(Cognitive Dysfunctions[Title/Abstract]) OR (Dysfunction, Cognitive[Title/Abstract]) OR (Dysfunctions, Cognitive[Title/Abstract]) OR (Cognitive Impairments[Title/Abstract]) OR (Cognitive Impairment[Title/Abstract]) OR (Impairment, Cognitive[Title/Abstract]) OR (Impairments, Cognitive[Title/Abstract]) OR (Cognitive Disorder[Title/Abstract]) OR (Cognitive Disorders[Title/Abstract]) OR (Disorder, Cognitive[Title/Abstract]) OR (Disorders, Cognitive[Title/Abstract]) OR (Mild Cognitive Impairment[Title/Abstract]) OR (Cognitive Impairment, Mild[Title/Abstract]) OR (Cognitive Impairments, Mild[Title/Abstract]) OR (Impairment, Mild Cognitive[Title/Abstract]) OR (Impairments, Mild Cognitive[Title/Abstract]) OR (Mild Cognitive Impairments[Title/Abstract]) OR (Cognitive Decline[Title/Abstract]) OR (Cognitive Declines[Title/Abstract]) OR (Decline, Cognitive[Title/Abstract]) OR (Declines, Cognitive[Title/Abstract]) OR (Mental Deterioration[Title/Abstract]) OR (Deterioration, Mental[Title/Abstract]) OR (Deteriorations, Mental[Title/Abstract]) OR (Mental Deteriorations[Title/Abstract]) OR (Amnestic cognitive impairment[Title/Abstract]) OR (Cognitive dysfunction[Title/Abstract]) OR (MCI [Title/Abstract]) OR (aMCI[Title/Abstract])
#3	#1 OR #2
#4	“Acupuncture”[Mesh]
#5	(Pharmacopuncture[Title/Abstract]) OR (acupuncture needle[Title/Abstract]) OR (electroacupuncture[Title/Abstract]) OR (electrical acupuncture[Title/Abstract]) OR (scalp acupuncture[Title/Abstract]) OR (manual acupuncture[Title/Abstract]) OR (ear acupuncture[Title/Abstract]) OR (meridian[Title/Abstract]) OR (acupuncture therapy[Title/Abstract]) OR (auriculotherapy[Title/Abstract]) OR (acupoint [Title/Abstract])
#6	#4 OR #5
#7	“Magnetic resonance imaging”[Mesh]
#8	(magnetic resonance image[Title/Abstract]) OR (NMR imaging[Title/Abstract]) OR (neuroimaging[Title/Abstract]) OR (MRI scans[Title/Abstract]) OR (functional MRI[Title/Abstract]) OR (fMRI[Title/Abstract]) OR (spin echo imaging[Title/Abstract])
#9	#7 OR #8
#10	#3 AND #6 AND #9

### 2.4 Eligibility criteria

#### 2.4.1 Participants

Participants were required to have a diagnosis of amnestic mild cognitive impairment (aMCI) based on established clinical diagnostic criteria, be aged between 40 and 85 years, with no restrictions regarding gender or educational background.

#### 2.4.2 Intervention

All therapeutic interventions involving percutaneous insertion of acupuncture needles to stimulate specific acupoints were eligible for inclusion, including manual acupuncture, abdominal acupuncture, auricular acupuncture, scalp acupuncture, and electroacupuncture. No restrictions were imposed on acupuncture prescription (e.g., acupoint selection), treatment duration, or session frequency, encompassing both standalone acupuncture and combination therapies. Additionally, stimulation parameters such as needle retention time, manipulation intensity, and stimulation frequency were not standardized.

#### 2.4.3 Comparison

Control groups received either sham acupuncture or guideline-recommended, acupuncture-excluded interventions such as health education, pharmacotherapy, cognitive-motor dual-task training, and non-invasive brain stimulation modalities including transcranial magnetic stimulation (TMS) and transcranial direct current stimulation (tDCS). If experimental groups received acupuncture combined with other therapies, control groups were required to incorporate the corresponding adjunctive therapies.

#### 2.4.4 Outcomes

(1) Primary outcomes: The primary outcomes comprised whole-brain functional imaging data obtained through resting-state functional magnetic resonance imaging (rs-fMRI). (2) Secondary outcomes: Validated instruments assessing clinical efficacy and cognitive performance were included, specifically the Montreal Cognitive Assessment (MoCA), Clinical Dementia Rating (CDR), Mini-Mental State Examination (MMSE), Alzheimer’s Disease Assessment Scale-Cognitive Subscale (ADAS-Cog), and Barthel Index (BI).

#### 2.4.5 Study type

All published randomized controlled trials (RCTs), prospective case–control studies, and observational studies addressing this topic were eligible for inclusion. Exclusion criteria encompassed reviews, animal studies, case reports, case series, and study protocols. Additionally, duplicate publications were excluded, with only the study possessing the largest sample size retained in cases of overlapping study populations or duplicated datasets.

### 2.5 Study selection

**Figure 1:**
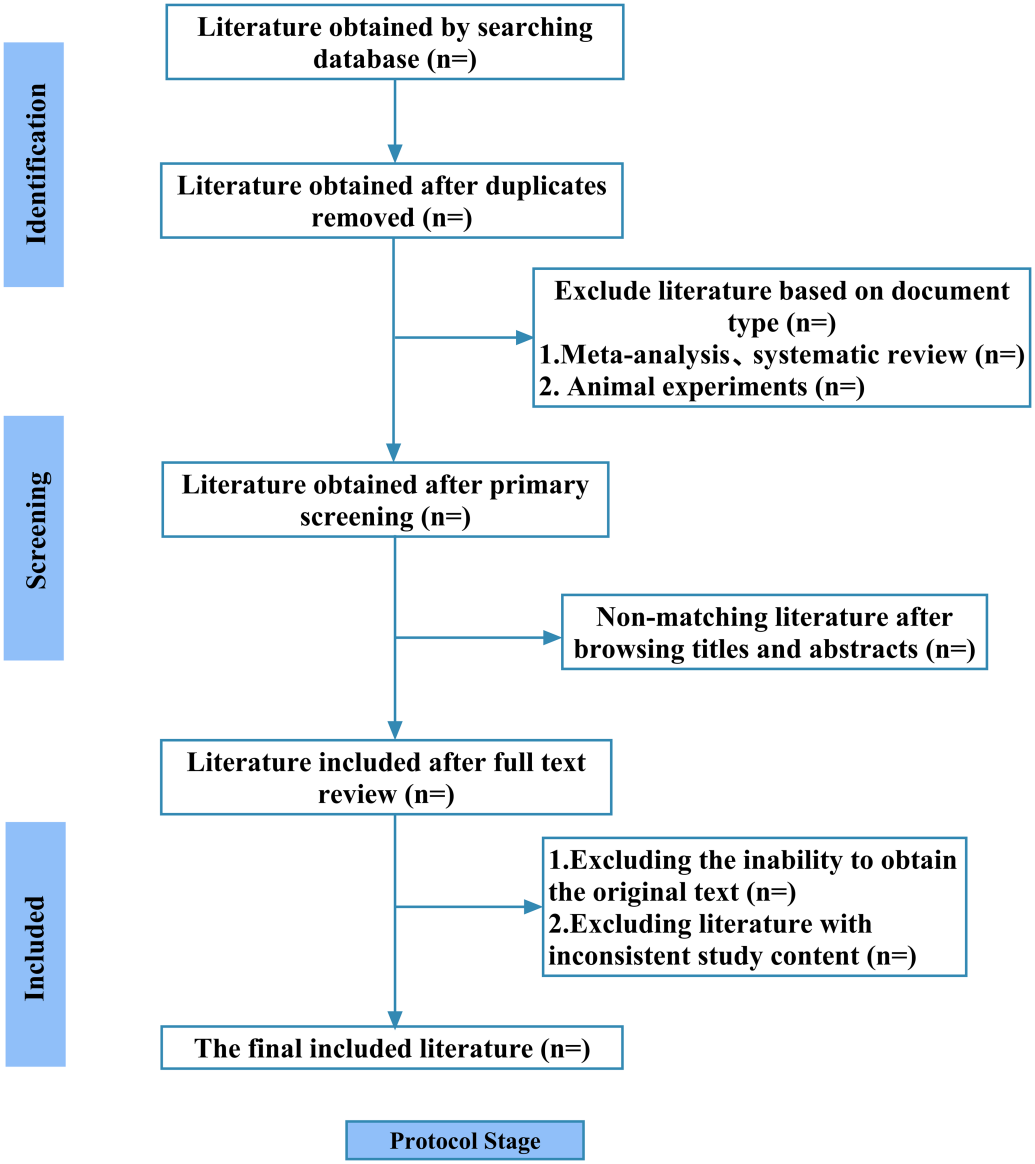
Literature screening process.

Two independent reviewers (DY and FQ) conducted the literature search and screening process. Identified records were managed using NoteExpress (version 4.1). Following deduplication, reviewers sequentially screened titles, abstracts, and keywords, followed by full-text assessments to determine study eligibility. Discrepancies were resolved through team consensus discussions. Exclusion rationales were systematically documented, with the screening workflow visualized in a PRISMA flow diagram, the literature screening process is shown in Figure 1.

### 2.6 Study extraction

A predefined data extraction form was developed in accordance with the Cochrane Handbook (version 5.1.0). Two designated data managers (DY and FQ) independently extracted relevant study characteristics using the standardized form. Extracted data included: (a) trial descriptors (principal investigator, publication year, sample size); (b) participant demographics and clinical profiles (disease duration, age, sex); (c) intervention protocols (acupuncture modality, needle retention time, treatment frequency, control group interventions); and (d) outcome measurements (neuroimaging parameters, secondary cognitive assessments, safety evaluations). Neuroimaging metrics encompassed the following parameters: (1) three-dimensional stereotactic coordinates (*X*, *Y*, *Z*) in either Montreal Neurological Institute (MNI) or Talairach reference spaces; (2) corresponding statistical parametric values (*t*-statistics, *z*-scores, or *p*-values); (3) voxel cluster extent; and (4) anatomical labeling of activated brain regions. Secondary outcome data quantifying treatment effects were extracted from immediate post-intervention assessments or evaluations conducted closest to treatment termination. During data extraction, studies with insufficient or missing data were identified, and corresponding authors were contacted via email to request supplementary information. Unresponsive studies were subsequently excluded, with exclusion rationales documented in the PRISMA flow diagram. An independent third researcher (TZ) then performed cross-validation of extracted data and adjudicated any identified discrepancies through standardized arbitration protocols.

### 2.7 Quality assessment

Building upon established methodological frameworks (22), we implemented a 13-item checklist to appraise the methodological quality of fMRI studies, with rigorous evaluation of sample size justification, methodological transparency, and comprehensive reporting of outcomes. Given the inclusion of both randomized controlled trials (RCTs) and observational studies, the Agency for Healthcare Research and Quality (AHRQ) checklist was selected to assess risk of bias across five critical domains: selection bias, performance bias, detection bias, attrition bias, and reporting bias (23). Two blinded independent assessors (TZ and YC) conducted quality evaluations, with discrepancies resolved through iterative Delphi consensus procedures.

### 2.8 Heterogeneity assessment

Heterogeneity was quantified by extracting MNI peak coordinates (Montreal Neurological Institute space) and calculating the standardized heterogeneity statistic *I*^2^, with *I*^2^ < 50% indicating low heterogeneity. Methodological variability in acupuncture protocols was further evaluated using the revised Standards for Reporting Interventions in Clinical Trials of Acupuncture (STRICTA) checklist to assess intervention-related heterogeneity sources (24).

### 2.9 Data synthesis

Meta-analyses of extracted fMRI data were performed using Seed-based d Mapping with Permutation of Subject Images (SDM-PSI) software (version 6.21; www.sdmproject.com). This analytic framework employed univariate voxel-wise testing with permutation-based methods to enhance statistical inference robustness (25). All subsequent analyses—including mean effect estimation, heterogeneity quantification, sensitivity testing, and meta-regression—were executed in strict accordance with SDM-PSI reference manual protocols.^1^ Results were tabulated in Microsoft Excel, with three-dimensional coordinate data mapped to MNI space using MRIcron (version 2). The statistical threshold was set at voxel-level *p* < 0.05 (FDR-corrected) with a minimum cluster extent of 10 contiguous voxels. Detailed descriptions of these procedures are provided in the SDM-PSI reference manual (see text footnote 1).

Clinical data pertaining to cognitive function scores were meta-analyzed using Review Manager 5.3 (The Cochrane Collaboration) and Stata 14.0 (StataCorp LP, United States). Between-study heterogeneity was assessed via Cochran’s *Q*-test (*χ*^2^) and quantified using the *I*^2^ statistic. Fixed-effects models were employed when Cochran’s *Q*-test yielded *p* > 0.1 with *I*^2^ < 50%, indicating nonsignificant heterogeneity. Conversely, random-effects models were implemented when *p* ≤ 0.1 or *I*^2^ ≥ 50% suggested substantial heterogeneity. Continuous outcomes were expressed as standardized mean differences (SMD) with 95% confidence intervals (CI), while dichotomous variables were analyzed using risk ratios (RR). Results were visualized through forest plots, with funnel plots generated to evaluate potential publication bias using Egger’s regression asymmetry test.

### 2.10 Assessment of publication bias

Publication bias will be assessed through visual inspection of funnel plot asymmetry and Egger’s regression intercept test. Asymmetry in the funnel plot suggests a higher likelihood of publication bias, which will be further confirmed using Egger’s test. A *p*-value <0.05 indicates the presence of publication bias, while a *p*-value ≥0.05 indicates the absence of such bias.

### 2.11 Sensitivity analysis

Sensitivity analyses will be conducted, including influence analysis and meta-analysis, by including or excluding trials with a high risk of bias to assess the robustness of the primary outcomes. Influence analysis qualitatively evaluates the impact of each individual study on the overall results by sequentially excluding one study at a time; meta-analysis quantitatively analyses robustness by excluding trials judged to have an overall high risk of bias.

### 2.12 Subgroup analysis

When significant between-study heterogeneity was detected (Cochran’s *Q* test: *I*^2^ ≥ 50%, *p* < 0.10), stratified subgroup analyses were performed based on predefined potential effect modifiers, which were limited to two categories: patient characteristics and intervention types. Subgroup analyses for patient characteristics primarily focused on age and education level, while those for intervention types mainly distinguished between different forms of acupuncture. Subgroup analysis by participant stratification was conducted to assess the influence of population-specific features on intervention outcomes, whereas analysis by intervention type aimed to elucidate differential efficacy among specific acupuncture modalities, such as manual acupuncture, electroacupuncture, and auricular acupuncture.

### 2.13 Grading the quality of evidence

The certainty of evidence was appraised using the Grading of Recommendations Assessment, Development and Evaluation (GRADE) framework (26). Evidence ratings were subject to downgrading based on five predefined domains: risk of bias, inconsistency, indirectness, imprecision, and publication bias. Final certainty levels were categorized into four tiers: high, moderate, low, and very low.

## 3 Discussion

The amnestic subtype of MCI (aMCI), characterized predominantly by memory impairment, constitutes a critical yet frequently overlooked transitional phase in the progression toward AD (27). Emerging fMRI evidence has corroborated these findings, demonstrating that cerebral aging follows a nonlinear trajectory characterized by accelerated progression commencing at age 44 and peaking at 67 years. Emerging evidence suggests that biomarkers may demonstrate limited sensitivity in detecting abnormalities during early-stage neurodegenerative processes of cerebral aging, posing challenges for detection during therapeutic interventions (28, 29). In contrast, functional magnetic resonance imaging (fMRI) enables earlier identification of neurofunctional alterations at this critical phase (30, 31). Current neuroimaging evidence reveals heterogeneous neural effects of acupuncture interventions in aMCI, manifested by inconsistent regional brain activation patterns. Such variability may stem from heterogeneity in experimental designs across studies. To address this knowledge gap, this systematic review and meta-analysis will integrate all functional magnetic resonance imaging (fMRI) studies on acupuncture for amnestic mild cognitive impairment (aMCI). By selecting resting-state fMRI metrics such as ALFF and ReHo, and combining voxel-wise pooled analysis via SDM-PSI, this study aims to pinpoint the brain regions within the default mode network that are modulated by acupuncture in aMCI patients. This design complements the limitations of existing research, which often focuses on isolated brain regions and lacks pooled analytical approaches, aiming to: (1) comprehensively synthesize neuroplastic signatures across brain networks; (2) identify potential neuromodulation targets; and (3) conduct subgroup analyses to investigate potential discrepancies between pooled effects and subgroup-specific outcomes. This methodological design emphasizes the methodological issue of heterogeneity in fMRI studies of acupuncture for aMCI. Subgroup analyses are employed to methodically identify sources of heterogeneity. Targeted subgroup analyses based on acupuncture intervention types and patient characteristics are designed to achieve “precise localization and stratified interpretation” of heterogeneity. Furthermore, STRICTA is applied to trace intervention-related heterogeneity, while the GRADE evidence grading system is introduced to assess outcome measures. By evaluating five domains—heterogeneity, risk of bias, indirectness, imprecision, and publication bias—the quality of evidence (high/moderate/low/very low) for outcomes is graded, thereby enabling a correlated interpretation of “heterogeneity and evidence quality.” This study conducts a voxel-wise meta-analysis of fMRI data. While focusing on the patterns of neural effects underlying acupuncture’s therapeutic efficacy, it also integrates the actual improvements in clinical cognitive function observed in patients across included studies, thereby examining the correlation between “alterations in neural mechanisms” and “alleviation of clinical symptoms.” Our findings are anticipated to elucidate the neurotherapeutic mechanisms underlying acupuncture-induced cognitive improvement in aMCI, delineate critical brain regions and activation patterns mediating these effects, and establish novel neuromodulation frameworks for targeted cerebral interventions in acupuncture-based aMCI management.

Current neuroimaging meta-analytic protocols predominantly employ voxel-wise coordinate mapping methodologies, utilizing three-dimensional Gaussian kernel smoothing and permutation-based thresholding to statistically converge reported stereotactic coordinates across studies (22). In this protocol, we implemented Seed-based d Mapping with Permutation of Subject Images (SDM-PSI) to conduct a systematic review and meta-analysis of fMRI studies investigating acupuncture interventions for aMCI. The present study protocol employs SDM-PSI as the core analytical tool, rather than traditional coordinate-based meta-analysis (CBMA) methods such as activation likelihood estimation (ALE). The primary rationale for this selection stems from the core logic of SDM-PSI, which integrates “voxel-wise effect size calculation” with “subject-based image permutation tests.” This approach is directly suited to the characteristics of resting-state data—specifically, differences in “regional brain activity intensity”—enabling more precise quantification of the magnitude of change in ALFF/ReHo following acupuncture intervention in patients with aMCI across studies. Furthermore, given the substantial heterogeneity present in fMRI studies of acupuncture for aMCI, SDM-PSI offers superior capacity for handling such variability. However, functional connectivity (FC) analysis was explicitly excluded from the pooled synthesis because FC examines interactions between distinct brain regions. Region-of-interest (ROI)-based approaches typically quantify correlations between the average time course of predefined ROIs and voxel-level time series across the whole brain or other ROIs, rather than providing specific spatial coordinates. In contrast, the coordinate-based meta-analysis (CBMA) conducted in this study fundamentally requires “spatial convergence”—all included studies must report coordinates so that algorithms can identify consistently activated brain regions. This methodological requirement is fundamentally incompatible with FC analysis, which does not provide the stereotactic coordinates necessary for spatial convergence in the CBMA paradigm. Therefore, FC analysis falls outside the scope of the present investigation. Our systematic literature search has revealed a critical gap in neuroimaging meta-analytic evidence, with fMRI-based synthesis studies remaining markedly underrepresented. This empirical lacuna substantiates the scientific imperative and methodological novelty of implementing our protocolized reporting framework. In summary, our primary objective is to establish robust evidence elucidating the neurotherapeutic mechanisms of acupuncture in aMCI through comprehensive synthesis of independent study outcomes. The anticipated findings are expected to reveal acupuncture-mediated reorganization of core cerebral networks in aMCI pathology, thereby providing empirically validated neural correlates to guide future investigations on neuromodulatory effects of acupuncture in early-stage neurodegenerative disorders.

## 4 Strengths and limitations of this study

(1) This study synthesizes findings from independent investigations to establish comprehensive evidence supporting the neurotherapeutic effects of acupuncture in aMCI.
(2) This study will rigorously adhere to methodological guidelines for systematic reviews and meta-analyses.
(3) This study employs seed-based d mapping with permutation of subject images (SDM-PSI) to conduct neuroimaging meta-analytic synthesis of neural effects and clinical cognitive functions, delineating acupuncture-induced neural modulation targets within cognition-associated cerebral regions. The findings further empirically validate the neurofunctional-clinical correlations underlying these cerebral reorganization patterns.
(4) Given the interdisciplinary nature of this study—which integrates acupuncture, aMCI, and resting-state fMRI techniques—the number of eligible studies meeting the stringent inclusion criteria may be limited. Should the final number of included studies be low, the statistical power of the meta-analysis would be insufficient to conduct reliable heterogeneity analysis, sensitivity analysis, and GRADE assessment. This could result in an inability to perform a meaningful meta-analysis (e.g., unstable effect size estimation, difficulty identifying consistently activated brain regions), and the conclusions would hold only exploratory rather than confirmatory value.

## Footnotes

1
www.sdmproject.com/manual/
